# Review of the Existing Translational Pharmacokinetics Modeling Approaches Specific to Monoclonal Antibodies (mAbs) to Support the First-In-Human (FIH) Dose Selection

**DOI:** 10.3390/ijms232112754

**Published:** 2022-10-22

**Authors:** Blaise Pasquiers, Salih Benamara, Mathieu Felices, Laurent Nguyen, Xavier Declèves

**Affiliations:** 1 PhinC Development, 91300 Massy, France; 2 Université Paris Cité, Inserm UMRS-1144, Optimisation Thérapeutique en Neuropsychopharmacologie, 75006 Paris, France; 3 Sanofi R&D, 91380 Chilly-Mazarin, France

**Keywords:** monoclonal antibody, pharmacokinetics, modeling, translational, first-in-human, PopPK, PBPK, mPBPK, ADA

## Abstract

The interest in therapeutic monoclonal antibodies (mAbs) has continuously growing in several diseases. However, their pharmacokinetics (PK) is complex due to their target-mediated drug disposition (TMDD) profiles which can induce a non-linear PK. This point is particularly challenging during the pre-clinical and translational development of a new mAb. This article reviews and describes the existing PK modeling approaches used to translate the mAbs PK from animal to human for intravenous (IV) and subcutaneous (SC) administration routes. Several approaches are presented, from the most empirical models to full physiologically based pharmacokinetic (PBPK) models, with a focus on the population PK methods (compartmental and minimal PBPK models). They include the translational approaches for the linear part of the PK and the TMDD mechanism of mAbs. The objective of this article is to provide an up-to-date overview and future perspectives of the translational PK approaches for mAbs during a model-informed drug development (MIDD), since the field of PK modeling has gained recently significant interest for guiding mAbs drug development.

## 1. Introduction

Antibodies, also known as immunoglobulins (Igs), are large proteins used by the immune system [1]. They can recognize an infectious microorganism, such as viruses, bacteria, or cells identified as pathogens. These proteins bind to their specific antigen and send a signal to the immune system for its’ destruction. Igs are composed of a base unit of two identical heavy chains and two identical light chains, held together by several disulfide bonds. In human, there are two types of light chains (κ and λ) and five types of heavy chains Ig (α, δ, ε, μ and γ) [2]. Igs are grouped into five classes according to the structure of their heavy chains: IgA, IgD, IgE, IgM and IgG. All approved therapeutic antibodies are IgGs. They represent the predominant class used due to their long half-life [3]. Of the five antibody classes, immunoglobulin gamma (IgG) is the most prevalent class in the serum and non-mucosal tissues [4]. IgGs are large biological molecule (~150 kDa) with high polarity [2]. IgGs can be divided enzymatically into two major structural units, a fragment antigen binding (Fab) and a fragment crystallisable region (Fc). The Fab recognizes the target while Fc binds to a range of cell-associated receptors such as Fc neonatal receptor (FcRn) and Fc gamma receptor (FcγR). The interaction with FcγR, expressed in various effector cells, can activate the host immune system [5].

Monoclonal antibodies (mAbs) are antibodies naturally produced by the same clone of activated B lymphocytes or plasma cells [6] and directed to recognize a specific target. Therefore, monoclonal antibodies have the ability to neutralize specific targets, with the help of the immune system including different mechanisms such as Antibody-Dependent Cell-mediated Cytotoxicity (ADCC) and/or Antibody-Dependent Cellular Phagocytosis (ADCP), and/or Complement-Dependent Cytotoxicity (CDC). The targets for therapeutic mAbs are often selected due to their involvement in a disease, such as cancer or inflammatory disease. Due to their physiological characteristics, therapeutic mAbs pharmacokinetics (PK) is different from traditional small weight therapeutic molecules in terms of absorption, distribution, and elimination.

mAbs are not absorbed orally (very limited bioavailability, typically, less than 1 or 2%) due to their large molecular size, poor lipophilicity and membrane permeability, and poor stability in gastro-intestinal fluids [2,7,8]. Thus, the preferred routes of administrations are subcutaneous (SC), intra-venous (IV) or intra-muscular (IM). The simplicity of subcutaneous administration (SC) and the growing interest in mAbs therapies for chronic diseases suggest their potential for SC administration. Pharmaceutical developers and clinicians have a common interest in transitioning from intravenous to SC mAbs because of the benefits of better patient compliance, including a better comfort enabling administration of the drug at home, and reduced costs to the health care system [9,10]. MAbs absorption within the systemic blood circulation is made via the lymphatic system and their SC bioavailability can vary between 20 and 95% [11,12,13]. The maximal plasma concentration (C_max_) is generally observed for a few days (1 to 8 days) after single dose SC administration [8,13].

In adults, the mAbs volume of distribution is limited to a few litres represented by the vascular and interstitial spaces of highly perfused and leaky tissues. This is due to their large size, low membrane permeability, and high solubility in aqueous fluids [2,14]. Therefore, the distribution in peripheral tissues protected by tight tissue barriers, such as the brain, is particularly low. Under certain circumstances, like tumours in oncology, tissue distribution of mAbs can be modified in pathological situations, for example when there is altered tissue barriers (e.g., inflammatory state, cancer) or when there is an over-expression of the target at the tumor site [15].

Contrary to small weight chemical drugs, mAbs do not undergo hepatic enzymes metabolism and/or biliary excretion and cannot be cleared in an intact form by renal glomerular filtration owing to their large molecular size. Two mechanisms of elimination are mainly described, a non-specific and a specific elimination. Non-specific degradation of mAbs occurs by pinocytosis following cellular endosomal uptake and subsequent lysosomal proteolytic degradation into amino acids or smaller peptides. Cellular uptake of antibodies is thought to occur mostly in endothelial and haematopoietic cells. Once mAbs are taken up into the endosomes, they can be protected from degradation by binding to the neonatal Fc receptor (FcRn). This receptor binds antibodies in a pH-dependent manner with higher affinity at a pH of 6 in the endosome than at the physiologic pH of ∼7.4 in plasma. Therefore, antibodies that are bound to FcRn in the endosome are released at neutral pH into the plasma, allowing recirculation of the antibody rather than lysosomal degradation. Due to this salvage mechanism, the FcRn receptor is a main driver for the long half-life of therapeutic mAbs [4]. At therapeutic doses, this mechanism of elimination by endosomal uptake and proteolysis is supposed to be unsaturable and is described as a linear clearance using first-order elimination constant [4,8,16].

The specific elimination is a target-mediated drug disposition (TMDD). This pathway of elimination begins with the binding of the mAbs to its target antigen. Then, the complex can be internalized in the cell and catabolized, or it can be degraded without cell internalization. The TMDD elimination is a typical feature of mAbs, and it is usually identifiable on the PK profile at the lowest concentrations of the drug, when the target is not saturated and when drug and target concentrations balance between them. This second mechanism of elimination leads to a non-linear elimination of the drug at low concentration (when the target is still not saturated). The linear pattern is observed either when mAbs concentration dominates over target concentration (i.e., target saturation situation) or when target concentration is much higher than mAb concentration [17].

The growing interest of model-informed drug development (MIDD) using pharmacometrics approaches has gained the acceptance of the regulatory agencies to improve drug development. Pharmacometrics is the science of using in silico models to understand and generate new knowledge of the underlying processes of the drug-patient-disease interactions by interlinking the biology, physiology, and pharmacology. It is essential to learn, describe, and predict pharmacokinetics (PK) and pharmacodynamics (PD) of therapeutics. PK and PD modeling and simulations during preclinical development is usually used to predict the safe starting dose in human, to identify the active dose, and to evaluate and anticipate the activity of the proposed clinical doses. However, translational mAbs modeling is known to be particularly challenging with several limitations. The main limitation is that mAbs PK will depend on its target and whether the target is present or not, as the expression of the target might vary widely between species. Does a TMDD mechanism in an animal predictive of a TMDD in a human and how can we translate it? And, if there is not a TMDD profile in an animal, how can we anticipate a potential TMDD in a human?

We will review in this article the different existing methods used in translational PK modeling for mAbs and discuss the current knowledge and limitations.

## 2. mAbs PK Modeling

mAbs PK modeling approaches are split into populational and physiologically based approaches:

- For the populational approach (popPK) using non-linear mixed effect modeling, models are data driven compartmental or semi-physiological (minimal PBPK) models. Usually, those models include parallel linear and non-linear components for the total clearance to account for linear and non-linear elimination.
- For the physiologically based pharmacokinetic approach (PBPK), models include physiologically based parameters to describe absorption, distribution, and linear elimination processes, and both target drug binding information to account for TMDD pattern if any.

### 2.1. Population PK Approach (NLME)

#### 2.1.1. Linear PK

##### Simplified Model

**Figure 1:**
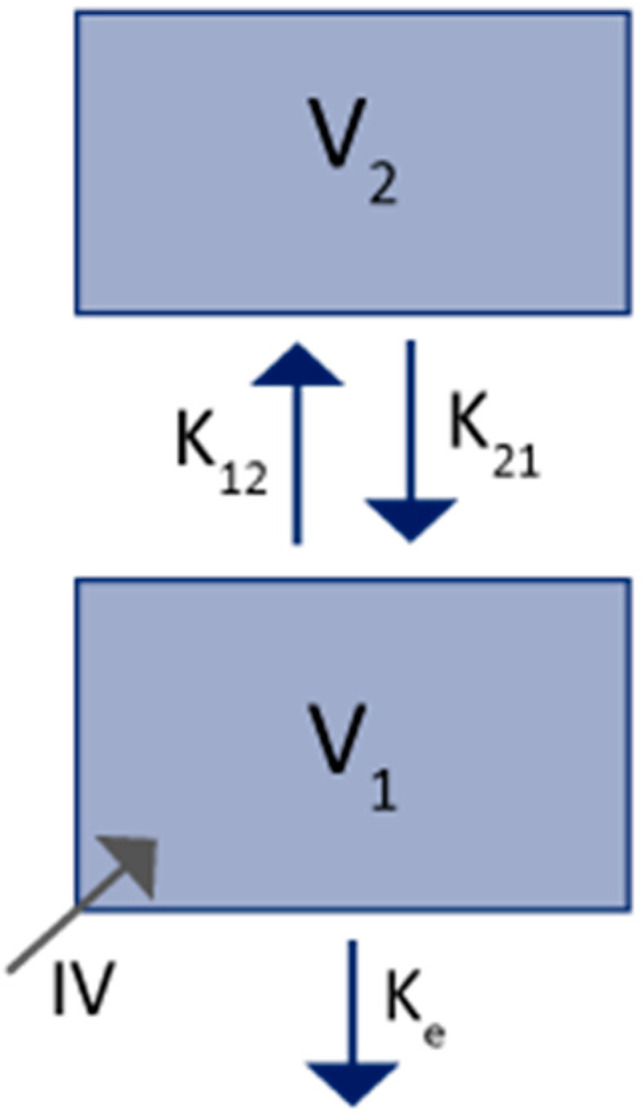
Simplified 2-compartments model for mAbs PK. V_1_: central volume; V_2_: peripheral volume; K_e_: first-order rate constant of elimination; K_12_: first-order rate constant of distribution from V_1_ to V_2_; K_21_: first-order rate of distribution from V_2_ to V_1_; IV: mAbs intravenous administration.

When its target is saturated, mAbs PK are relatively simple and therefore easy to model in comparison to small molecules. In fact, simplified models for mAbs are well-known and are generally represented by a 2-compartments model with linear distribution and elimination (Figure 1). Fronton et al. showed in a thorough study that this model structure provided results very close to those obtained from the PBPK model and consistent with the experimental data. However, she could not distinguish which tissues were physiologically described by each of the two compartments and results were similar with a clearance from the central (V_1_) and/or peripheral compartment (V_2_) [18]. We represented the model with elimination from the central compartment in Figure 1.

Volumes of distribution of mAbs are smaller compared to small molecules and close to blood and interstitial spaces volumes [8] and the PK distribution behaviour is well conserved between mAbs. The central compartment is commonly identified as the plasma volume and the peripheral compartment as the interstitial space. The half-life is long (several days) and depends mainly on the affinity with the FcRn which protects the mAbs from the non-specific elimination. It is well recognised that the extent of the linear clearance is correlated with the FcRn affinity: the higher the affinity, the lower the clearance of mAbs.

Some models describe the mAbs PK more physiologically as minimal PBPK (mPBPK) or full PBPK models.

##### mPBPK Model

**Figure 2:**
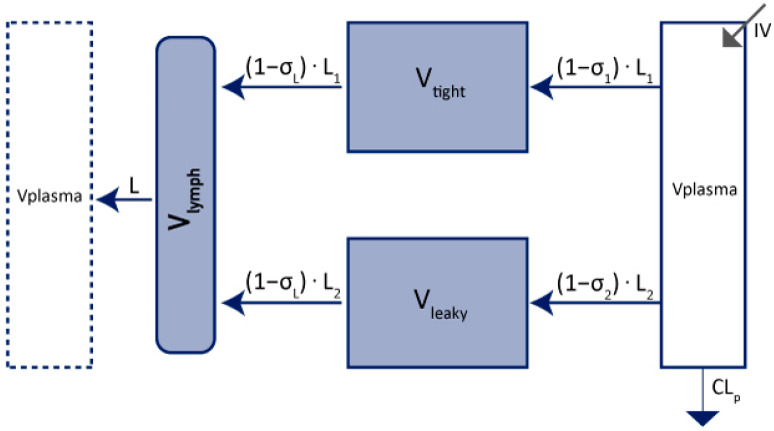
Representation of 2nd generation of mPBPK adapted from Cao et al. [19]. V_plasma_: plasma volume; V_tight_: tight tissues volume; V_leaky_: leaky tissues volume; Clp: clearance from plasma volume; L: total lymph flow; L_1_: lymph flow for V_tight_; L_2_: lymph flow for V_leaky_; σ_1_: vascular reflection coefficient for V_tight_; σ_2_: vascular reflection coefficient for V_leaky_; σ_L_: lymphatic capillary reflection coefficient; V_lymph_: lymph volume; IV: mAbs administration by IV. V_tight_ = 0.65 × ISF × K_p._ V_leaky_ = 0.35 × ISF × K_p._ L_1_ = 0.33 × L. L_2_ = 0.67 × L. ISF: total system interstitial fluid; K_p_: available fraction of ISF for mAb distribution.

In 2013, Cao et al. published a mPBPK, which considers some physiological aspects of the mAbs PK without requiring as much physiological information as a full PBPK model [19]. The model describes a drug administered by IV route in the central compartment. It can be distributed through two groups of tissues according to their vascular endothelium structures. We can distinguish tissues that have continuous capillaries (tight compartment) and tissues with non-continuous capillaries (leaky compartment). Muscle, skin, adipose, and brain are described to correspond to the tight compartment, while other tissues such as liver, kidney, and heart correspond to the leaky compartment (Figure 2). The σ_1_ and σ_2_ parameters are vascular reflection coefficients and σ_L_ is the lymphatic capillary reflection coefficient.

V_lymph_ is the lymph volume. It is supposed to be equal to the blood volume. As in the 2-compartment model, mAbs can be eliminated in the plasma as well as in the tissue’s compartments. Figure 2 represents the elimination from the plasma compartment. Minimal PBPK model includes more physiological description of the distribution process of mAbs; they are parameterized using more model parameters compared to simplified 2-compartments model, however, they rely on non-estimated parameters fixed to physiological values.

**Figure 3:**
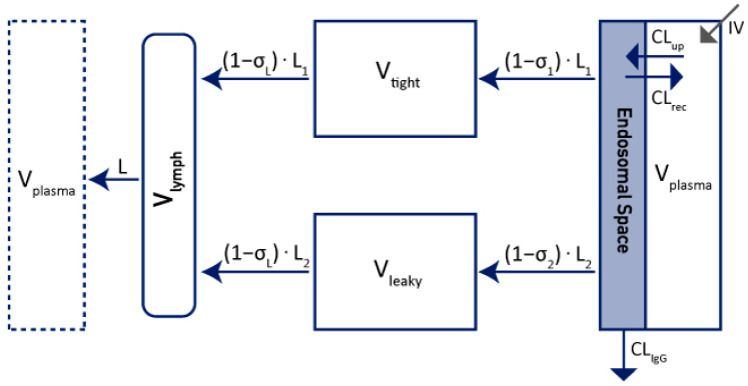
Extended mPBPK model with endosomal compartment adapted from Maas et al. [20]. V_plasma_: plasma volume; V_tight_: tight tissues volume; V_leaky_: leaky tissues volume; L: total lymph flow; L_1_: lymph flow for V_tight_; L_2_: lymph flow for V_leaky_; σ_1_: vascular reflection coefficient for V_tight_; σ_2_: vascular reflection coefficient for V_leaky_; σL: lymphatic capillary reflection coefficient; V_lymph_: lymph volume, Cl_up_: rate from V_plasma_ to endosomal space; Cl_IgG_: clearance from endosomal space; Cl_rec_: recycled from endosomal space to V_plasma_; IV: mAbs administration by IV. Cl_rec_ = Cl_up_ − Cl_IgG_.

Maas et al. improved this model in 2018 [20] by including an endosomal compartment in parallel with the plasma compartment in order to explain the changes in mAb-FcRn affinities in the endosomal space due to the transit time and the pH difference (see Figure 3) [20,21].

The endosomal space is parallel to the plasma compartment and has a negligible volume. An antibody is taken up into the endosomal space at a rate of Cl_up_ and cleared out of the body (Cl_IgG_). IgG is recycled back into the plasma at a rate of Cl_rec_. The elimination from the plasma compartment is assumed to be negligible in this modeling context.

#### 2.1.2. Target-Mediated PK

The models described above only consider the linear non-target dependent elimination of mAbs. When the target is not saturated by the mAbs concentrations, a TMDD profile is visible which corresponds to the specific elimination of the mAb bound to its target.

Therefore, it is interesting to know the level concentrations of saturation of the targets by the mAbs. As mentioned by Oitate et al. in 2011, mAbs are split in two categories, depending on their target [22]. Generally, mAbs which target membrane-bound antigen will saturate their target at higher concentrations than mAbs targeting soluble antigen. In this second case, TMDD profiles are generally not visible because it is happening at a concentration below the limit of quantification, while for membrane-bound targets, a TMDD profile can appear at higher concentrations and doses [22,23]. In some cases mAbs targeting soluble targets with a high turnover and endogenous levels, can have a target-mediated kinetics [24].

The TMDD profile is more complex to capture by modeling. It can be described by the Michaelis–Menten equation as its simplest way in addition to the linear elimination (which still represents the non-specific elimination) or by a more complex TMDD model including the binding equations between mAb and its target, the free target, and target-mAbs complex dynamics.

**Figure 4:**
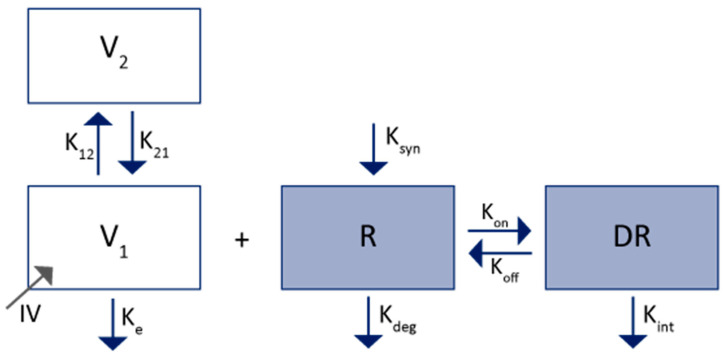
Pharmacokinetics model of a TMDD adapted from Mager and Jusko [25]. V_1_: central volume; V_2_: peripheral volume; K_e_: first-order rate constant of elimination; K_12_: first-order rate of distribution from V_1_ to V_2_; K_21_: first-order rate of distribution from V_2_ to V_1_; R: receptor-target concentration; K_syn_: zero-order synthesis rate of the target; K_deg_: first-order degradation rate of the target; K_int_: first-order internalization rate of the DR complex; K_on_: first-order association rate of the mAb with the target; K_off_: first-order dissociation rate of the mAb with the target; DR: drug-receptor complex concentration; IV: mAbs administration by IV.

Mager and Jusko were the first to develop this kind of model in 2001 (adapted in Figure 4) [25]. The TMDD model includes a target turn-over model with synthesis (K_syn_) and degradation (K_deg_) rates, the drug-target binding equilibrium process with association (K_on_) and dissociation (K_off_) constants, and the complex elimination with the rate of internalization/degradation (Kint). To account for the non-target specific elimination of the free drug, a first order elimination constant (K_el_) from the central compartment is used.

Currently, a large panel of TMDD models are described in the literature with different approximations, from a full TMDD model to a simplified model with parallel linear and non-linear elimination (MM).

**Figure 5:**
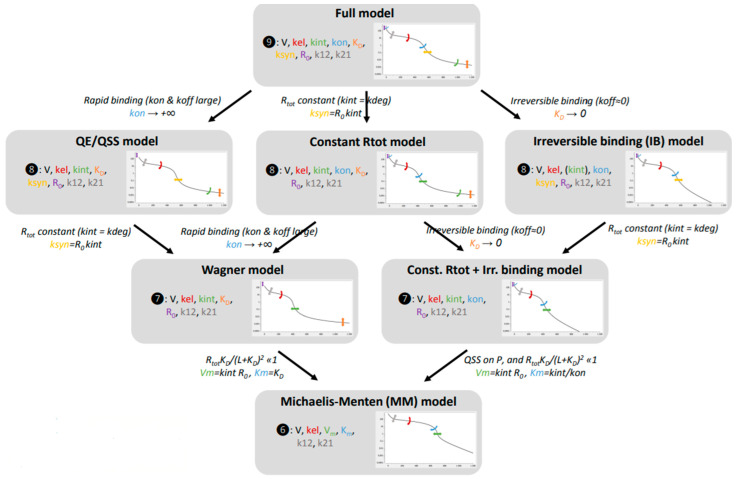
TMDD modeling hierarchy reprinted with permission from Lixoft^®^ [26]. Different models’ approximation to describe a TMDD profile. QE/QSS: quasi-equilibrium or quasi-steady state approximation; V: central volume; K_el_: clearance rate; K_int_: first-order internalization rate of the complex mAb-target; K_on_: first-order association rate of the mAb with the target; K_d_: first-order dissociation constant of the mAb with the target; K_syn_: zero-order synthesis rate of the target; R_0_: initial target concentration; K_12_: first-order rate of distribution from V_1_ to V_2_; K_21_: first-order rate of distribution from V_2_ to V_1_; K_m_: Michaelis–Menten constant.

Lixoft^®^ described these different models as visible in the figure below (Figure 5).

The full model is the more physiological one. However, it is also the more challenging one as it requires rich data observations for estimating all model parameters to avoid identifiability issues. In fact, in addition to the values of V, K_12_, K_21_, K_el_, in the linear part, the full model needs to estimate K_int_ (internalization of the complex mAb-target), K_on_ (first-order association rate of the mAb with the target), K_d_ (dissociation constant of the mAb with the target), K_syn_ (zero-order synthesis rate of the target), and R_0_ (initial target concentration). Some of these values can be fixed to either known physiological values (e.g., R0, Kdeg) or values determined by in-vitro experiments (e.g., K_on_, K_off_, K_d_). Several model simplifications based on assumptions were also proposed to reduce the number of model parameters that need to be estimated. Further details on the parameterization and assumptions of these simplified TMDD models are documented in the Gibiansky et al. publication [27]. The three major hypotheses used in these models are:

- No difference between complex and target elimination rate;
- A rapid binding of the mAb with its target;
- An irreversible binding of the mAb with its target.

The QE/QSS model, Wagner model, and MM model seem to be the most used as the hypothesis of a rapid binding is the most realistic [28].

**Figure 6:**
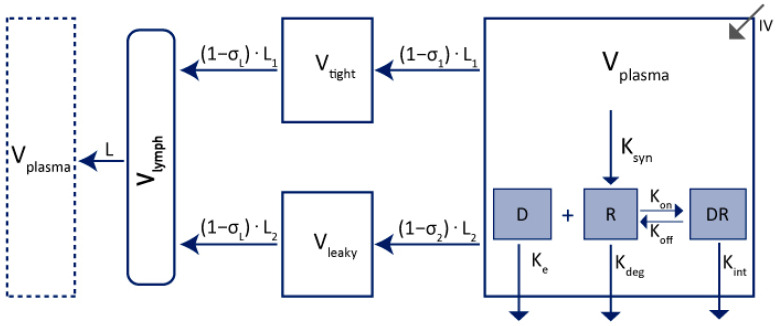
mPBPK of a mAbs including TMDD adapted from Pawaska et al. [29]. V_plasma_: plasma volume; V_tight_: tight tissues volume; V_leaky_: leaky tissues volume; K_e_: first-order rate constant of endogenous elimination of drug from plasma volume; L: total lymph flow; L_1_: lymph flow for V_tight_; L_2_: lymph flow for V_leaky_; σ_1_: vascular reflection coefficient for V_tight_; σ_2_: vascular reflection coefficient for V_leaky_; σ_L_: lymphatic capillary reflection coefficient; V_lymph_: lymph volume; D: Drug concentration, R: Receptor-target concentration; DR: drug-receptor complex concentration; K_syn_: zero-order synthesis rate of the target; K_deg_: first-order degradation rate of the target; K_on_: first-order association rate of the mAb with the target; K_off_: first-order dissociation rate of the mAb with the target; K_int_: first-order internalization rate of the DR complex; IV: mAbs administration by IV.

These elimination models can be included in a simple 2-compartment model as well as a mPBPK model (Figure 6).

### 2.2. PBPK Approach

One approach that could be used to identify possible targets for drug discovery programs would be to use full PBPK modeling. It is able to generate predictions of doses in order to achieve a targeted receptor occupancy for mAbs that exert their effect according to the binding with their targets [30]. The use of full PBPK models represents an attractive approach for plasma and tissue PK prediction, as they are able to integrate knowledge across various levels, from the anatomical space to the molecular level [31]. PBPK models are a platform that has become prevalent in recent years. They were initially developed to describe small molecule PK, then they were subsequently extended to mAbs. The first PBPK model developed for mAbs was described in 1986 [32] and included six tissues: lung, intestine, liver, spleen, kidney, and skin. They were divided into three layers: capillary plasma, interstitial space, and cellular space [32]. Although this model was based on adjustment of several parameters, it was able to properly characterize plasma and IgG tissue concentration over time after administration in mice. MAbs disposition was described through the distribution based on the tissue uptake of antibodies that is done via convective transport. Modeling analyses suggested that more than 98% of mAbs enters tissue via a convective pathway [33].

Since this first model, several predictive PBPK models of mAbs disposition were published [21,34,35,36]. Despite the moderate confidence in the mAbs PBPK modeling due to uncertainties in parameters related to TMDD and to FcRn binding, these models were able to describe relatively well the time course of drug exposure [37].

**Figure 7:**
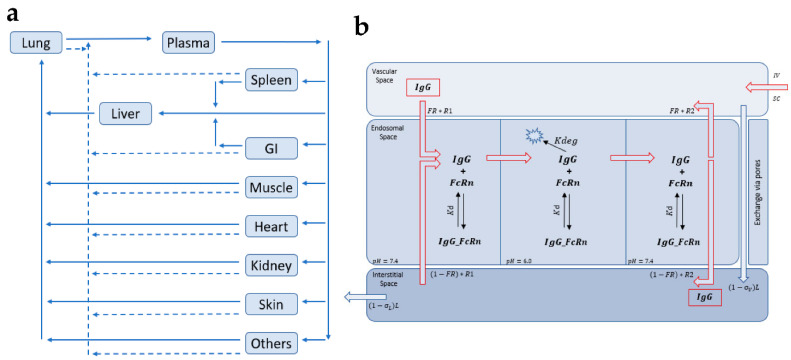
(**a**) PBPK model of IgG disposition where the major organs are included in the model, and the various compartments are connected by plasma (solid arrows) and lymphatic flow (dashed arrows). Each tissue within this model is divided into sub-compartments [34] (**b**) Intra-tissue compartmental model of IgG (mAb) disposition.

The model in Figure 7a was used as a basis for most of the recent PBPK models for mAbs [34]. Tissue compartments were divided into vascular, endosomal, and interstitial sub-compartments where the FcRn-mediated protection of mAbs is typically described as occurring within the endosomes of the vascular endothelium and is described either via equilibrium or kinetic binding parameters depending on the pH [34].

Each organ is divided into three sub-compartments representing the vascular, endosomal, and interstitial space as schematized in Figure 7b. (L) is the lymph flow, (R1) is the uptake rate of the IgG in the endosomal compartment via fluid-phase endocytosis, ($\sigma_{\;v}$) and ($\sigma_{\;I}$) are the vascular and lymph reflection coefficients, Kd is the dissociation coefficient for the binding of IgG with FcRn, Kdeg is the first order degradation rate of the unbound IgG, (R2) is the recycling rate of the unbound IgG, and (FR) is the bound recycled fraction [34].

## 3. mAbs PK Translation

As for the modeling, the translation depends on if there is the presence of a TMDD profile or not. For a mAb without TMDD, the PK translation from pre-clinical to clinical development after IV administration is well described in the literature. Several methods are described, and they performed well to predict human PK. In this paper, we will describe a few of the most used methods. On the one hand, allometry and scaling time-invariant methods are the oldest and the more empirical of them, and the half-life method is a new empirical approach. On the other hand, translation using mPBPK and full PBPK approaches are more recent and based on physiological knowledge. All the methods have some advantages and some limitations, however, they also depend on the animal species used for scaling. Majority of articles in the literature agree with the fact that the monkey is the best specie to translate mAbs PK to humans [10,22,38,39,40,41]. In fact, while for small molecules multiple-species scaling are preferred, for mAbs, single-specie scaling seems to be a better solution for two reasons:

- Central volume of distribution is limited to blood and interstitial volume for each species, without large variability, so it simplifies the volume translation;
- Linear elimination mainly depends on the affinity of the mAb with the FcRn, and cynomolgus FcRn has a similar binding affinity for human IgG as human FcRn does, whereas murine FcRn appears to have a much higher affinity to human IgG than the human FcRn [8,42].

Therefore, PK translation of mAbs is preferentially obtained from the cynomolgus monkey, due to its similarity with human in several aspects. Indeed, therapeutic mAbs are generally cross-reactive between cynomolgus and human targets, and the binding affinities are in the same order of magnitude. However, the cynomolgus model presents some limitations for translational purpose since anti-drug antibodies (ADA) are frequently observed in monkey and because there is no disease bearing model for this specie as opposite to mouse model (e.g., absence of tumor-bearing or genetically modified disease models).

A transgenic mice model that expresses human FcRn was developed (Tg32-mouse) and has several advantages. It permits rationalising the use of monkeys in drug development and allows for reducing the apparition of anti-drug antibodies (ADA) in some cases in preclinical development [43]. This apparition is species-dependent and not representative of what will happen in humans (especially for fully humanised mAbs) [44,45].

Betts et al. (2018) confirmed that the Tg32-mouse model was very encouraging, as it predicted well the human PK [46]. Frances et al. showed in 2021 that the Tg32-mouse could be even more predictive than monkeys in some case [47]. It is supposed to be a rare case, but it is important to mention to improve in the future the use of these Tg32-mouse models.

Another advantage of using the mice model is the possibility to integrate the targeted disease in the animal, like a tumor implanted, or mice genetically modified to express human biomarkers. It allows for the description of the impact of the disease on the PK and describes the pharmacodynamics in terms of the efficacy of the drug in animals. For ethical reasons and technical difficulties, diseases cannot be reproduced in monkeys.

### 3.1. Population PK Translation

#### 3.1.1. Translation of Linear PK

##### Allometry

The allometry scaling is based on the hypothesis that a parameter can be scaled only by the weight. The equation used is:

$$P_{human}=P_{animal}\times {\left(\frac{BW_{human}}{BW_{animal}}\right)}^{\alpha }$$

where *P* represents a *PK* parameter (clearance, volume…), *BW* is the body weight and α the allometric exponent.

For a small molecule, standard allometric exponent values are 0.75 for clearance and 1 for volumes of distribution. For mAbs, other values are mentioned in the literature. The values recovered from the literature are listed in the Table 1 below (non-exhaustive list).

Zheng et al. (2012) tried to translate the PK from minipig to human using allometric scaling with good results [50]. They concluded that the minipig could be a great alternative of the monkey model without TMDD profiles. Additionally, even if the classic allometric exponents determined for small molecules (0.75 for clearances and 1 for volumes) are still used for mAbs translation [51], it seems that the range of allometric exponents for mAbs are around 0.85 for clearance and 1 for the volume of distribution. A small distinction can be made between the mAbs with a soluble target (clearance: around 0.85) and with a membrane-bound target (clearance: around 0.9). This scaling seems to be reliable for the different molecules tested with linear pharmacokinetics. Haraya et al. distinguished the exponent for Cl/F (after SC administration) and Cl (after IV administration). As F after SC administration seems to be lower in humans than in monkeys [52], the allometric exponent needed to be slightly higher for the translation of Cl/F [10]. Moreover, multiple species allometric scaling method can be adjusted with correction factors such as maximum life span potency (MLP) or brain weight (BrW) [38,41,53]. However, results are limited even if, in some cases, it can improve the prediction. It can also predict worse human PK, especially when the Cl estimated without correction factors was already underestimated [38,41,53]. These methods originate from small molecules and were tested for mAbs but are scarcely used because of different PK-related physiological characteristics between species as mentioned above (e.g., FcRn differential affinity, no cross-reactivity…). Wang also proposed to use antigen concentration (AC) as a correction factor, dividing the Cl by AC before scaling the product by allometry. He found this factor more relevant than MLP or BrW [53], according to the following equation:

$$\frac{Cl}{AC}=a\times BW^{b}$$

##### Scaling Time-Invariant

In the scaling time-invariant approach, the scaling is directly made on the concentration-time profiles. It is based on two points:

- as for the allometric scaling, the concentrations are correlated to the body weight;
- and time equivalency between two species is also correlated to the body weight [54].

Same allometric exponents as for allometric translation of PK parameters are used (*α_volume_* and *α_clearance_*) [55].

MAbs serum concentration-time in linear phase can be transformed by this time-invariant scaling method using two equations [54,56,57,58]:

$$Time_{human}=Time_{animal}\times {\left(\frac{BW_{human}}{BW_{animal}}\right)}^{\alpha_{volume}-\alpha_{clearance}}$$

$$Concentration_{human}=Concentration_{animal}\times \frac{Dose_{human}}{Dose_{animal}}$$

where *Time_human_* is pharmacokinetic time in human; *Time_animal_* is pharmacokinetic time in animal; *Concentration_human_* is mAb serum concentration in human; *Concentration_animal_* is mAb serum concentration in animal; *Dose_human_* is dose in human PK study (mg/kg); *Dose_animal_* is dose in animal PK study (mg/kg).

A scaling exponent of 0.85 for clearance and 1 for volume of distribution was used by Gupta et al. in 2016 [57]. These values could be adapted with the allometric scaling seen previously. With these concentration-time profiles predicted in human, a new model can be built. The advantage of this approach is mainly to predict the entire PK profile at the expected dose and not only the unique PK parameters [55].

##### Half-Life Method

In 2020, Nakamura et al. proposed a new empirical method of human PK prediction from non-human primates (NHP) data. The aim of this method was to gain in efficiency during the drug development of linear mAbs and to take part of the 3Rs initiative rules (refinement, reduction, and replacement) [23].

**Figure 8:**
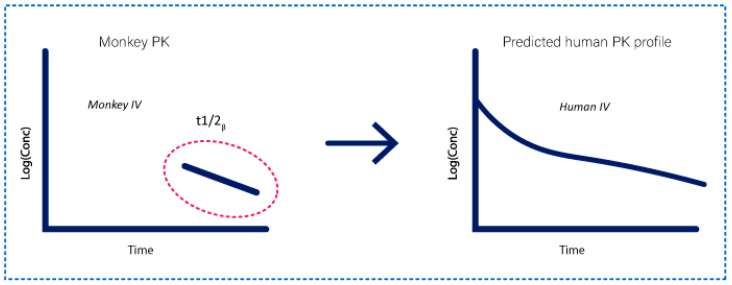
Representation of the half-life scaling method adapted from Nakamura et al [23]. The terminal monkey half-life (t1/2_β_) of a mAb is sufficient to predict the IV PK profile in human with the half-life method.

The principle is to only use the half-life of the mAb in NHP to predict the linear PK in human as visible in Figure 8.

For this, Nakamura et al. obtained geometric mean (GM) values of the PK parameters from 24 mAbs in human modelled by a simple 2-compartment model. They explained that human values of distribution parameters (V_1_, K_12_, K_21_, V_dss_; see Figure 1) were not necessary to extrapolate from pre-clinical data to humans, as the distribution was very low for all mAbs with a small variability. They are fixed to the GM value in mAbs (K_12_: 0.275 day^−1^, K_21_: 0.355 day^−1^, V_1_: 45.1 mL/kg, and V_dss_: 81 mL/kg). K_12_ and K_21_ have more variability, but Nakamura et al. showed that the ratio between these two values was constant: K_21_ = 1.32 × K_12_.

Therefore, human prediction only depends on the translation of K_10_ from NHP. This scaling starts with a prediction of human elimination rate constant using body weight based allometric scaling.

$$\beta_{human}=\beta_{NHP}\times {\left(\frac{BW_{human}}{BW_{NHP}}\right)}^{X}\;$$

*β* is elimination rate. *BW* is the body weight. If no specific body weight information is found in the literature, a weight of 70 kg for humans, 3.75 kg for cynomolgus monkeys, or 6.0 kg for rhesus monkeys can be used. *X* is the scaling exponent for rate constant. It can be fixed generally to −0.15 for mAbs.

The second step is to calculate *K*_10_:

$$K10_{human}=\beta_{human}\times \left(\frac{Vdss_{human}}{V1_{human}}\right)\;$$

The values used for this equation are the *β_human_* calculated at the first step and the GM of V_dss_ and V_1_.

Based on 19 mAbs, Nakamura et al. showed that this method gave comparable results than those obtained from an allometric scaling on a simple 2-compartment model. However, this method reduces the number of data needed from pre-clinical studies to obtain the only t1/2_β_. It could be useful in the future of mAbs development [23].

Nakamura et al. continued in 2021 their research on the half-life method to extrapolate human PK profiles from human FcRn (hFcRn) transgenic mice (Tg32 model) [59].

The same previous values were used to describe the human distribution phase (K_12_, K_21_, V_1_) and they found a correlation between the half-life in hFcRn mice and in humans based on 10 mAbs.

$$t1/2\beta_{human}=1.89\times t1/2\beta_{Tg32}+7.18$$

$$K10_{human}=1.8\times \left(\frac{Ln\left(2\right)}{t1/2\beta_{human}}\right)\;$$

They showed that this correlation was good when excluding mAbs with an AUC_β_ (AUC related to the elimination phase) lower than 70% of the AUC_inf_. In these cases, multiple other factors than FcRn recycling are expected to impact the clearance of the mAbs, like presence of ADA (Anti-drug antigen), isoelectric point, and local charge patches [8,59,60].

The second paper of Nakamura et al. allows for the improvement in respect to the 3R rules (Replacement, Reduction, Refinement).

##### mPBPK Scaling

The scaling from a minimal PBPK is a mix between an allometric scaling and the use of physiological values. The values of V_tight_, V_leaky_, V_lymph_, L_1_, L_2_ can be obtained from the literature [61,62,63,64]. Each mAb is often assumed to have the same vascular reflection coefficients among species for both tight tissues (σ_1_) and leaky tissues (σ_2_) [64]. However, these values can also be fixed to a median value of mAbs. The lymphatic capillary reflection coefficient (σ_L_) is usually assumed to be 0.2 for both cynomolgus and human [63,64]. Plasma volume can be translated by allometric scaling from monkey or fixed to a physiological value.

Yuan et al. summarised physiological values that can be useful in the translation to humans (Table 2) [65]. They refer to a mPBPK model as presented in Figure 2.

The last value to translate is the clearance, and this value can be scaled with the same rules as seen in the “allometric scaling” section. In the improved model including endosomal space, the affinity to FcRn, and endosomal transit time T(w) can be translated using experimental values to improve mAbs clearance across mice, monkeys, and humans [20]. For example, the rate of endosomal uptake (Cl_up_) was estimated across species and scaled well with bodyweight, and the affinity to FcRn has been identified as an important factor for increasing the persistence of mAbs.

##### Scaling of SC “Absorption”

When considering PK translation after a SC administration, another parameter to translate from animal to human is the absorption in terms of rate and extent. The SC absorption is generally split in two parameters, the first-order rate of absorption (K_a_) and the bioavailability (F). Richter et al. showed in 2020 that mAbs SC absorption was generally faster in animals than in humans due to their physiology [12,50,66]. One of the translational methods for K_a_ could be to scale the parameters from the monkey as the other parameters with an allometric exponent of −0.25 [29,67], even if the C_max_ can be regularly over-predicted with this exponent and the translation is considered as unreliable by some papers [63,66]. In other papers, K_a_ was assumed to be the same in humans and in monkeys [51]. Human bioavailability is also often fixed to the same value as the bioavailability in monkeys [51,63,67]. Singh in 2021 also proposed the time-invariant scaling including absorption by SC administration [63].

Zheng et al. tried in 2012 to scale the absorption from the minipig, as similarities between minipig and human skin and lymph architecture were demonstrated [50,68,69]. The absorption rate and bioavailability correlation between human and minipig were weak in this work. However, it could be interesting to work with more data on this potential correlation, especially as the bioavailability was the same in humans and minipigs for rituximab and trastuzumab [70]. Recently, Richter et al. showed that administration of tocilizumab by SC behind the minipig’s ears was the best location to translate the bioavailability in human [66], without confirmation in other mAbs. Whereas Zheng et al. administered the drug in the inguinal or scapular area [50]. This can explain the weak correlation obtain by Zheng et al.

Another alternative for the translation in humans could be to fix the K_a_ values to the mean of mAbs values in humans. This method was used by Haraya et al. based on the geometric means of the K_a_ from 19 mAbs (0.287 day^−1^) [10,49]. Based on 6 mAbs, Dirks found a K_a_ value around 0.217 day^−1^ (0.132 to 0.48) for a human [14].

Bown et al. also proposed a prediction of human F (bioavailability) using an in vitro model called Scissor. Scissor allows the prediction of F of mAbs with a good correlation. Their study was validated with only 8 mAbs, but could be interesting to improve further SC human PK prediction [71].

Overall, the subcutaneous absorption translation in mAbs is poorly documented and with limited reliability, and none of these methods seems to be preferentially used.

#### 3.1.2. Translation of Target-Mediated PK

As shown before, the translation of the distribution and linear elimination was correctly documented in the literature with good methodologies and results. However, one of the main issues in the translation of mAbs PK occurs in case of non-linearity and TMDD profile because the translation should include the association with the target. Concerning the target, differences between animals and humans can be large. The principal issue is to have the human target in the animal model. The monkey was preferentially used for the similarity of its physiology with the human. However, even if the human target is present in the animal, several points can have an impact on the TMDD profile: the antigen target kinetics, the association constant of the drug with the target, and the target density [23]. All these elements are species-dependent and are particularly challenging to translate. If mAbs have a poor affinity for the animal target in comparison to the human target, the use of surrogate can be necessary. A surrogate needs to have a similar pharmacokinetics and pharmacodynamics in the studied animal than the lead mAb in human [72]. It needs to bind the animal target with the same affinity as the candidate binds the human one [56]. Their affinity for FcRn needs to be the same too. These similar affinities will help to anticipate the PK (especially the TMDD part) and the PD in human. However, the use of a surrogate increases the complexity and the challenges to translate the PKPD from animals to humans [72]. Few methods were tested to translate TMDD profile in humans with limited results. First, the same methods as for mAbs without TMDD can be used to scale up the linear PK part of mAbs including a TMDD. For example, Singh used the QE/QSS model). To translate the linear parameters part, he used an allometric scaling [67]. This method is largely used and found in each paper with a mAbs TMDD [29,51,63,73].

Then, for the other target-dependent parameters, we can distinguish these methods by the type of PK model:

- Empirical approaches:

For a translation with a simple 2-compartment model including MM elimination, hypotheses are to consider the K_m_ equivalent in humans and in monkeys, and to scale Vmax with the same coefficient as linear Cl [39,47,74]. The analysis performed on six mAbs with TMDD profile showed, as expected, that the translation was better for doses corresponding to the linear PK pattern than doses corresponding to the non-linear pattern. Singh et al. choose to translate K_m_ from monkey to human by allometry with a exponent value of 1 [63]. Another empirical method reviewed by Kamath [8] was the scaling by species-invariant time method. However, the conclusion was the same as for the allometric scaling. This method is acceptable for the linear PK part but needs to be used with caution for TMDD part.

- Semi-physiological approach:

When Kamath wrote his review in 2016, it was the beginning of the semi-physiological translational modeling for mAbs [8]. This method has been better described since; however, some limitations remain. The semi-physiological approach is split in two types of models, TMDD integrated into a simple popPK model as described by Mager et al. in 2001 [25], or, more recently, into a minimal PBPK model as represented by Pawaskar et al. in Figure 6 [29]. Scaling of the TMDD parameters was equivalent for both methods. Nowadays, the scaling of this model widely relies on a mix of allometric scaling values, values reported from monkey model, and in vitro values.

We reported in the Table 3 below the different hypotheses made from recently published papers.

In conclusion, it is difficult to highlight a clear methodology for the translation of the TMDD profile. For now, the translation of non-linear mAbs TMDD is case-dependent.

Singh et al. in 2021 tried to compare the use of the three classic methods of translation: by allometric scaling of the PK parameters of a classical 2-compartmental model, by species time-invariant method, or by scaling of a mPBPK [63]. This work was performed in five mAbs. To describe the non-linear PK, they used a Michaelis–Menten elimination model in parallel to a linear elimination. For the 2-compartment model and the mPBPK model, they scaled V_max_ and K_m_ with allometric exponents of 0.85 (as for the clearance) and 1, respectively. They concluded that the best method to translate mAbs with TMDD was to use a mPBPK model then a species time-invariant method, rather than a simple compartmental allometric scaling. Parng et al. in 2018 showed that an anti-TFPI mAb had better results in predictions and were obtained with a model including the target in the model (TMDD model with QSS approximation) than with a simple MM elimination [51]. Even if these works need to be completed by further studies, they conclude that semi-physiological models seem to be more robust to translate PK from animal to human, whether for the description of the linear part using mPBPK models of Cao et al. or for the non-linear PK (including the target in a TMDD model) [19].

### 3.2. PBPK Translation

#### 3.2.1. mAbs Disposition

The first PBPK model to incorporate FcRn-mediated IgG recycling in all tissues was developed in mice [34]. Simulations using only physiological parameters without adjustments were able to generate good plasma and tissues PK predictions in wild-type mice (Swiss-Webster mice) and in FcRn-knockout mice (without FcRn expression). This demonstrated the utility of this model structure and mechanisms for the prediction of mAbs non-specific distribution and elimination [34].

The scaling of mAb PK in the absence of target-mediated elimination is relatively simple in PBPK due to the conserved nature of processes controlling non-specific elimination of mAbs. Often it can be described as using the same model structure as for preclinical species [31]. However, target expression can contribute to TMDD in monkeys and in humans, and these values are not easily measured in these large species.

Targets expression data are very rarely reported quantitatively for humans, particularly in healthy tissues. Nevertheless, semi-quantitative measures of protein expression are available in the literature [62]. Using an algorithm linking immunohistochemistry (IHC) scores to interstitial receptor concentrations, a PBPK model to predict non-linear mAb disposition in plasma and in tissue was scaled to monkeys and humans [35,62]. This approach demonstrated its ability to make reasonable translational PK predictions of several antibodies with linear PK using a strategy similar to that described for mice [75]. Even if the limitations of this approach are recognized for the TMDD part, the use of IHC scores to make predictions for tissue target concentrations in addition to the use of key parameter values from literatures [62] represents a potential for mechanistic TMDD description and translation in PBPK models [62]. In this context, only a few translational PBPK models for mAbs were described in the literature. Glassman and Balthasar scaled up a model in humans in 2016 for four mAbs (cetuximab, figitumumab, dalotuzumab, and trastuzumab) with TMDD profiles [35]. Predictions were accurate for three of the four mAbs, especially in the TMDD range of concentrations. The two determinant parameters in the prediction of TMDD were the target accessibility and expression and the rate of its turnover. Only figitumumab was not predicted well, however it has been reported as an atypical anti-IGF1R mAbs with an unclear mechanism at the origin of the PK differences with other anti-IGF1R. This can explain the inability of the model to predict its PK well.

#### 3.2.2. mAbs Subcutaneous Absorption

The predominant route of mAb absorption in SC administration is via the lymphatic routes due to the high molecular weight and the high hydrophilicity of mAbs [52]. This absorption is FcRn-dependent and saturable [9]. Essential parameters are represented by:

- the rate of fluid from injection site through the lymphatics to the circulation (tissue lymph flow, reflection coefficient, and blood rate);
- the expression of FcRn in cells present in lymphatics routes [76].

Unfortunately, values of these parameters have not been reported with high confidence in the literature, limiting the development of PBPK absorption model for mAbs [76]. SC absorption is often described through administration of mAb to the skin interstitial space. From this space, mAb can leave the tissue via the lymphatics and the lymph nodes to the circulation. For example, an empirical function allows the description of SC bioavailability. It relates the mAb fraction returning from lymph node to plasma with the FcRn binding affinity [21]. This approach has shown good predictability in mice [21]. However, the prediction of bioavailability in large species (e.g., monkeys and humans) with this simple function is poorly reliability due to the potential inter-species differences in the FcRn capacity [76].

### 3.3. Dealing with ADA

We distinguish mainly four types of monoclonal antibodies:

- murine antibodies (-omab)
- chimeric antibodies (-ximab)
- humanized antibodies (-zumab)
- human antibodies (-mumab).

**Figure 9:**
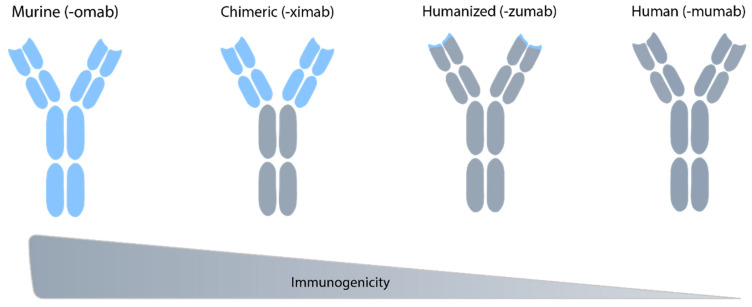
Representation of the four types of monoclonal antibodies adapted from Santos et al. [78]. Blue: murine part of the mAb; grey: humanized part of the mAb. The more the mAb is humanized, the less it is immunogenic.

While murine antibodies contain exclusively murine amino-acid sequences, the three other types are more humanized (60% for the chimeric, 90% for the humanized, and 100% for the human antibodies). In theory, the amino-acid sequence of an antibody has a large role in the immunogenicity of the monoclonal antibody, however immunogenicity is a complex issue which depends on multiple factors and is still poorly understood. The less it is humanized, more it is immunogenic for a human. Conversely, a highly humanized antibody can be more immunogenic for an animal than for a human (Figure 9) [77].

The interpretation of PK data with observed ADA is another challenging point during the mAbs PK translation. In fact, immunogenicity will induce the development of Anti-Drug Antibodies (ADA), which, by linking with the mAb, can increase the mAb elimination. ADA alters mAb PK and mAb efficacy consequently [79,80]. ADA can appear from 7 to 14 days after the first administration of the mAb; they can be transient or persistent [43]. Pre-existing ADA can also be found due to similarities with natural antibodies or previous administered mAbs [81]. The ADA observed in animals are generally not predictive of immunogenicity in humans and the presence of ADA makes it difficult for PK interpretation. It is necessary to distinguish a non-linear elimination due to an ADA effect or due to TMDD. This made possible by measuring ADA using specific assay methods in animal and human settings. For interpretation purposes, a simple way is to exclude all PK data, which follows ADA production in pre-clinical development [82]. An alternative way is to model ADA clearance on top of non-specific and target-specific clearance [83]. Modeling ADA effect on the PK is increasingly used in industry to keep maximum information for PK/PD assessment. The difficulty in ADA modeling is that ADA effect can be vary considerably as it may affect mAbs PK with different extents or may not have any impact at all [56].

## 4. Discussion

The mAbs dose selection during the drug development is a crucial point, especially for the first-in-human dose. Generally, higher therapeutic doses are required to effectively suppress soluble targets compared to membranous ones. However, Tang et al. also showed in a large review the importance of the mechanism of action (immunomodulatory function, membrane signaling suppression, or soluble target neutralization) during the therapeutic dose selection rather than the nature, turnover, and abundance of the target [84]. Even if the dose selection depends on many factors, the PK translation and prediction, and the affinity to the target remain key issues.

Our review of the literature clearly indicates that the translation of linear mAbs PK (distribution and linear elimination) is generally well described. Empirical methods extrapolated from small molecules models are largely described with good prediction results and are easy to set up. There is a consensus on the scaling of the simplified 2-compartment model and the minimal PBPK model. The allometric scaling can be used with some small adjustments of the exponent values depending on the case, with an exponent of around 0.85 for the clearance and 1 for the central volume of distribution. Physiological values used to scale mPBPK are also well characterized in the literature. Other empirical methods have been used, like time-invariant scaling or the half-life method; these methods are straightforward and easy to set up. Whereas the use of monkeys is largely preferred to predict human mAbs PK. The more recent half-life method would allow the reduced use of such animals during drug development, even to replace the use of monkeys by human FcRn transgenic mice. Use of PBPK in this context of linear PK could bring more physiological information especially for PK predictions in tissues. However, its use is not generalized yet and seems to be not always useful as empirical methods allow the prediction of human PK reasonably well.

However, it should be highlighted that these translation methods are not adapted for TMDD prediction. This issue can be extended to the translation of PKPD modeling, where the impact of a target and the dynamics of the target need to be quantitatively assessed. The description of TMDD can be performed using models (2-compartment and mPBPK) described for the linear mAbs PK adding TMDD equations, using either Michaelis–Menten equation or more mechanistic TMDD models including the dynamics of the target and the drug-target complex. These TMDD equations can also be included in a full PBPK model. Moreover, recent Quantitative System Pharmacology (QSP) are increasingly used and integrate drug mechanism of action with cellular interactions and communications.

QSP modeling allows for a better description of cells dynamics and their interaction with the mAb, thus enables prediction of TMDD in a mechanistic way [85]. Such approaches are still exploratory but could be very useful to integrate complex mechanism of action, especially for mAbs in immunology or immune-oncology and for the new generations of bi- or multi-specific antibodies scaffolds. These physiological approaches (full PBPK-TMDD and QSP) are not routinely used; they need to be improved and better described. There is still a need to better understand the mechanism of action of the drug and to also obtain physiological and experimental information in humans and animals (e.g., about the target) in order to scale PK from animal to human.

Another issue during the translation of mAbs PK from animal to human is the potential development of ADA. ADA can largely impact the PK of mAbs, increasing the clearance of the drug. It is necessary to distinguish a non-linearity in the PK profile due to a TMDD mechanism from an ADA effect. This is possible by the quantification of ADA during drug development and accounting for ADA clearance in the PK model. However, ADA development is species-dependent, and its impact can vary dramatically. To date, no model has been published to predict the translation of ADA and this issue is a key point during animal studies for interpretation purposes in the case of the presence of an ADA. The non-linearity of mAbs PK can also be induced by time-dependency (non-stationarity). This non-stationarity in mAbs PK is mainly found in chronic disease, like tumors, because it is often explained by the course of the disease. This point was not discussed in the article because PK time-dependency is generally not observed during animal studies. Indeed, drug treatments in animals are shorter than in patients, and disease models in animal do not model the course of the disease well. However, it is important to anticipate this phenomenon in patients PK profiles.

Translation of non-linear mAbs PK is thus complex due to a combination of several issues. In conclusion, translation of TMDD remains challenging and there is no standardized method to solve this issue. Whereas first translation models were mainly empirical to consider only the linear part of the PK, inter-species extrapolation in mAbs PK has recently improved but still requires more complex models, including as much mechanistic aspects as much as possible. The most recent models still in development are full PBPK and QSP models to develop physiology in the model. Although the ability to predict non-linear patterns of mAbs should be improved by the development of these models, such approaches require a clear understanding of the mechanism of action and access to physiological information on the species (e.g., targets expression, location, dynamics, and regulations) and drug-dependent parameters (e.g., in vitro drug-receptor binding affinity and complex dynamics).

## Figures and Tables

**Table 1:** Review of allometric exponents to translate linear PK of mAbs toward humans in literature.

Author	Animal Model	Method	Clearance	Volume
Betts (2018) [46]	monkey	14 mAbs	0.81 (Cl) Q (0.57)	1
Oitate (2011) [22]	monkey	24 mAbs	0.79 (soluble) 0.96 (membrane)	1.12 (soluble, V_ss_) 1 (membrane, V_ss_)
Deng (2011) [38]	monkey	13 mAbs	0.85	1
Dong (2011) [39]	monkey	10 mAbs	0.75 No advantages of 0.85 or 0.9	1
Wang (2010) [48]	monkey	12 mAbs/Fc fusion proteins	0.8	
Ling (2009) [40]	monkey	14 mAbs	0.85 (soluble) 0.9 (membrane)	
Haraya (2017) [49]	monkey	17 mAbs	0.8 (Cl) 0.75 (Q)	1 (V_c_) 0.95 (V_p_)
Haraya (2021) [10]	monkey	13 mAbs	0.9 (Cl/F)	1.1
Betts (2018) [46]	tg32-mouse	8 mAbs	0.9 (Cl) Q (0.67)	1
Zheng (2012) [50]	minipig	9 mAbs	0.98	

Cl: Clearance; Q: Inter-compartmental clearance; Cl/F: apparent oral clearance; V_c_: central volume; V_p_: peripheral volume; V_ss_ = Steady-state volume; soluble refers to a soluble target; membrane refers to a membrane-bound target.

**Table 2:** Models parameters for a 70 kg human [65].

Parameters	Definition	Human Value
V_p_	Volume Plasma	2.6 L
K_p_	Fraction of ISF for IgG1 distribution	0.8
ISF	Total system interstitial fluid	15.6 L
V_tight_	Volume in tight tissue	8.1 L
V_leaky_	Volume in leaky tissue	4.4 L
σ_1_	V1 vascular reflection coefficient for tight tissues	0.945 *
σ_2_	V2 vascular reflection coefficient for leaky tissues	0.697 *
V_Lymph_	Lymph volume	5.2 L
L	Lymph flow	0.121 L/h
L_1_	V1 lymph flow	0.0399 L/h
L_2_	V2 lymph flow	0.0810 L/h
σ_L_	Lymphatic reflection coefficient	0.2

* Mean from 10 mAbs values.

**Table 3:** Review of TMDD scaling from monkey in literature.

Author	Kdeg	R0	Kd	Kon	Koff	Kint
Singh [67] (2014)	-experimental in vitro value -or else sensitivity analysis within a range (same value as monkeys or allometric scaling)	monkey value, adjusted by in-vitro values	monkey value, adjusted by in-vitro values	NA	NA	-experimental in-vitro value -or else sensitivity analysis within a range (same value as monkeys or allometric scaling)
Luu [73] (2012)	based on the half-life of the target (same for monkey and human)	monkey value	NA	experimental in vitro value	experimental in vitro value	Experimental in-vitro value
Pawaskar [29] (2021)	literature value	literature value in human	NA	in-vitro value	in-vitro value	allometric scaling
Parng [51] (2018)	monkey value	monkey value	experimental value in vitro	NA	NA	monkey value

NA: non applicable. Kdeg: first-order degradation rate of the target; R0: initial concentration of the target; Kd; dissociation constant of the mAb with the target; Kon: first-order association rate of the mAb with the target; Koff: first-order dissociation rate of the mAb with the target; Kint: first-order internalization rate of the DR complex.

## Data Availability

Data is contained within the article.
